# Injectable Hydrogels for Nervous Tissue Repair—A Brief Review

**DOI:** 10.3390/gels10030190

**Published:** 2024-03-09

**Authors:** Gladys Arline Politrón-Zepeda, Gabriela Fletes-Vargas, Rogelio Rodríguez-Rodríguez

**Affiliations:** 1 Ingeniería en Sistemas Biológicos, Centro Universitario de los Valles (CUVALLES), Universidad de Guadalajara, Carretera Guadalajara-Ameca Km. 45.5, Ameca 46600, Jalisco, Mexico; gladys.politron3496@alumnos.udg.mx; 2 Departamento de Ciencias Clínicas, Centro Universitario de los Altos (CUALTOS), Universidad de Guadalajara, Carretera Tepatitlán-Yahualica de González Gallo, Tepatitlán de Morelos 47620, Jalisco, Mexico; ana.fletes3623@alumnos.udg.mx; 3 Departamento de Ciencias Naturales y Exactas, Centro Universitario de los Valles (CUVALLES), Universidad de Guadalajara, Carretera Guadalajara-Ameca Km. 45.5, Ameca 46600, Jalisco, Mexico

**Keywords:** regenerative medicine, injectable hydrogels, tissue engineering, nervous tissue, neurodegenerative diseases

## Abstract

The repair of nervous tissue is a critical research field in tissue engineering because of the degenerative process in the injured nervous system. In this review, we summarize the progress of injectable hydrogels using in vitro and in vivo studies for the regeneration and repair of nervous tissue. Traditional treatments have not been favorable for patients, as they are invasive and inefficient; therefore, injectable hydrogels are promising for the treatment of damaged tissue. This review will contribute to a better understanding of injectable hydrogels as potential scaffolds and drug delivery system for neural tissue engineering applications.

## 1. Introduction

Tissue engineering is a highly multidisciplinary field that aims to substitute, repair, and replace damaged tissue in neurologic diseases, combining scaffolds, cells, and bioactive molecules, both in vitro and in vivo [1,2,3,4]. The combined effect of these three components offers advanced opportunities for tissue regeneration [5]. Scaffolds can supply the basic physicochemical, structural, biomechanical, and biological environment for cellular function and neo-tissue formation [4,6].

Nerve tissue repair is a fundamental field of research in tissue engineering because the degenerative process in the injured nervous system begins after damage to the plasma membrane, which acts as a barrier. Subsequently, cell death induced by necrosis or apoptosis occurs, leading to tissue loss. Therefore, there has been great interest in proposing innovative alternatives to restore nerve tissue [7,8,9]. The main challenge of tissue engineering is the functional repair of tissue injuries caused by wounds, diseases, infections, and ischemia by creating a suitable scaffold biomaterial that mimics natural tissue [10,11,12]. Scaffolds should mimic native tissue and have controllable biodegradability, appropriate mechanical properties, and superior biocompatibility that are suitable for cell growth, proliferation, adhesion, and differentiation [13,14,15,16]. Thanks to this, cells are able to sense and respond to the topography and stiffness of scaffolds [17]. The mechanical properties of scaffolds are essential for neural tissue engineering, as the brain is the softest organ in the body. Scaffolds must mimic the mechanical properties of the brain with adequate stiffness to allow for cell attachment [18]. For example, the mechanical stress experienced by the neuronal membrane along the scaffold surface interface dictates axonal growth and directionality [19]. Previous reports indicated that cortical neurons cultured in hydrogels exhibit superior cell survival and neural extension when the elastic modulus of the hydrogel approaches that of the softer extracellular matrix.

The surfaces of scaffolds can be modified using bioactive molecules such as short peptide sequences, laminin, fibronectin, vitronectin, and long chains of extracellular matrix proteins that enable and promote cell proliferation and adhesion [20]. Topography is crucial in favoring neurite attachment. For example, nanostructured surfaces that mimic the architecture of the extracellular matrix can favor cell propagation, proliferation, adhesion, neurite extension and branching, migration, and electrical signal transmission, while topography influences neural stem cell differentiation [18]. Previous studies have shown that neural cells can align and elongate in the direction of aligned nanofibers more clearly than those grown on random nanofibers [17]. Furthermore, contact guidance, which describes the propagation of cells in response to contact with surface topography, is a crucial factor for neural regenerative medicine. It can be achieved by multidimensional structures, ranging from planar structures to three-dimensional scaffolds [17].

**Figure 1:**
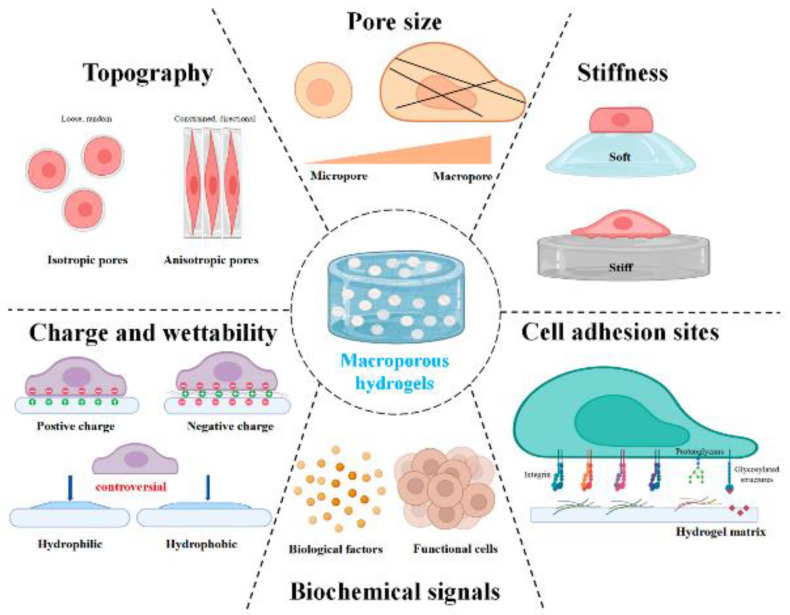
Porous microstructure of the hydrogels in cell behavior. Reprinted from Ma et al. [21].

The polymeric structure of a scaffold must immobilize molecules within the core of the material, such as antibiotics, anti-inflammatory drugs, growth factors, and neurotrophic factors [18]. In addition, the scaffold must have an adequate topography, porosity, and pore size for cell adhesion and for the diffusion of residues, nutrients, and growth factors into the polymeric porous structure [20] (Figure 1). If the scaffold is biodegradable, it will not need to be surgically removed, as it will be absorbed by the neural tissue. Therefore, biodegradable scaffolds aid nerve cell proliferation before being dissolved by the body while healing occurs [20]. Ideally, these scaffolds must possess electrical conductivity, facilitating interneuronal communication [18].

Hydrogels are a promising class of biomaterials produced by natural and synthetic polymers with high water content, high porosity, and mechanical properties like those of native tissue [22,23,24]. Thanks to this, hydrogels can be structurally and mechanically adjusted to mimic various tissues and contribute to regeneration through mechanical support of the tissue [25].

In recent years, studies have focused on the development of hydrogels as biodegradable scaffolds with suitable properties for tissue engineering and regeneration of the central nervous system. For example, injectable hydrogels can be injected into target areas with low invasiveness and mimic various aspects of the central nervous system [26,27].

Several review articles about injectable hydrogels for nervous tissue repair have been previously reported [26,28,29,30,31,32,33,34,35,36,37,38,39,40,41,42]. Recently, Gao et al. [29] published a review article describing injectable hydrogels in nerve repair and regeneration after ischemic stroke. However, the authors only focused on in vitro studies, which do not fully represent the applicability of scaffolds in neuronal tissue engineering applications.

Thus, this review provides a brief overview of recent advances in injectable hydrogels for the in vivo repair of nerve tissue derived from brain, peripheral nerve, and spinal cord injuries.

## 2. The Nervous System

The function of the nervous system is to monitor and control most automatic processes and activities. It comprises the central nervous system and the peripheral nervous system, which is classified into somatic and autonomic systems. The nervous system is a system with specific limitations, such as a low capacity for the proliferation and regeneration of neurons damaged during neurodegenerative pathologies, such as traumatic injuries, Parkinson’s disease, and Alzheimer’s disease [43].

The regenerative capacity of the central nervous system is limited by neurological conditions that trigger a cascade of events leading to secondary neuronal degeneration and death, offering limited therapeutic options to patients [26]. Therefore, there is a clinical need to develop therapeutic strategies for intractable neurological disorders. Nerve tissue engineering is a diverse biomedical field that combines experimental and computational neuroscience, clinical neurology, biomaterials science, and nanotechnology to address neurological diseases from a new perspective [44,45,46].

The type of cells and their extracellular matrix are the key components that determine their functions and properties, such as cell proliferation, migration, and differentiation [47]. The extracellular matrix of the central nervous system is composed of an extracellular matrix formed by fibrous proteins such as elastin or collagen embedded in an amorphous gel formed by non-fibrous components, usually glycoproteins formed by a core protein, which include a highly organized scaffold that is connected to the surface of the cells by adhesive molecules [48,49].

### 2.1. Diseases of and Damage to Nervous Tissue

Central nervous system injuries may be due to trauma (e.g., traumatic brain injury, traumatic spinal cord injury, stroke) or degeneration (e.g., multiple sclerosis, Alzheimer’s, Parkinson’s) [26]. These pathologies cause severe neurological dysfunction due to neuronal cell death and axonal degeneration. Neurons have little capacity to regenerate their axons and rebuild neuronal circuits lost after injury, because damage to the plasma membrane exposes the internal environment to extrinsic factors derived from the damaged axons. As a result, repressive growth molecules are secreted by glial cells, forming scars, so the tissue cannot regenerate [7,50,51].

#### 2.1.1. Spinal Cord Injury

Spinal cord injury is one of the most common and serious traumatic diseases; most cases occur in young adults, who face enormous physical challenges, with no treatment currently available. After suffering from contusion, compression, or traumatic accidents, the epicenter region of the spinal cord undergoes a complex pathological change, including primary and secondary injury. The former directly results in tissue damage and neural cell death [52]. The poor regenerative capacity of the human central nervous system results from the need to maintain functional stability. This is a biological advantage for a complex nervous system built on billions of interneuronal connections established during growth and development and is in contrast with the peripheral nervous system, which effectively regenerates after many types of injury. Nevertheless, neuronal failure of spinal cord injury results from many factors, including glial and stromal scarring formed after injury, which blocks axon growth and increases the inhibitors associated with myelin debris and proteoglycan deposition in the lesion environment [53].

#### 2.1.2. Traumatic Injury

Traumatic injuries to the nervous system can cause different types of structural damage; there are multiple consequences following traumatic injury, such as diffuse axonal injury, brain contusion, hematomas, skull fractures, etc., with both the central and peripheral nervous systems being affected [54].

Spinal cord injury is a very debilitating condition, which can result in partial or total paralysis, and places a considerable economic, physical, and emotional burden on patients and their families [55,56]. The current treatment for spinal cord injury includes the surgical decompression of the injured segments and the administration of steroids, which neutralize acute inflammation and decrease swelling to further reduce compression on any remaining neurons [57,58].

#### 2.1.3. Peripheral Nerve Injury

Patients with peripheral nerve injury develop painful neuropathy and neuroma, poor sensation, weakness, and paralysis following traumatic, nontraumatic, and iatrogenic experiences. These pathologies are derived from motor and sensory axon damage and loss of function [59]. Although the peripheral nervous system is more easily regenerated than the central nervous system, the clinical repair of peripheral nerve injury is still not satisfactory [60].

Hydrogels have become a popular material in tissue engineering due to their great potential to face those challenges. A critical characteristic in trauma injuries is the disconnection of axon pathways [61]. Cell-based therapies have shown great promise by targeting damaged axonal pathways. Still, the strategies proposed are not designed to restore long-distance axons; novel strategies enhance axons’ intrinsic ability to regenerate and create a permissive environment for axonal outgrowth [8,62,63]. Transplantable “scaffolds” have recently been used to facilitate axon regeneration. Although this is a promising strategy, the results of in vitro tests show that the number and length of the axons that grow along the scaffolds have been limited [64,65,66,67].

#### 2.1.4. Brain Injury

The brain is a complex tissue of the central nervous system which has the function of integrating and regulating signals and information in the nervous system along with the spinal cord [40]. Patients with traumatic brain injury are susceptible to permanent neurological deficits, which influence their daily lives [68]. Traumatic brain injury can be classified into primary injury and secondary injury. Primary injury can cause damage by direct mechanical forces in short periods of time, leading to hemorrhages, focal cerebral contusions, traumatic axonal injury, cerebral edema, and so on. Secondary injury occurs after an initial injury and is distinguished by the extension of damage from the center of the trauma [68].

During ischemic damage, the brain’s blood supply is reduced, leading to the loss of neuroglial cells, tissue framework, and extracellular matrix [69].

## 3. Hydrogel Scaffolds Used in the Regeneration of the Nervous System

**Figure 2:**
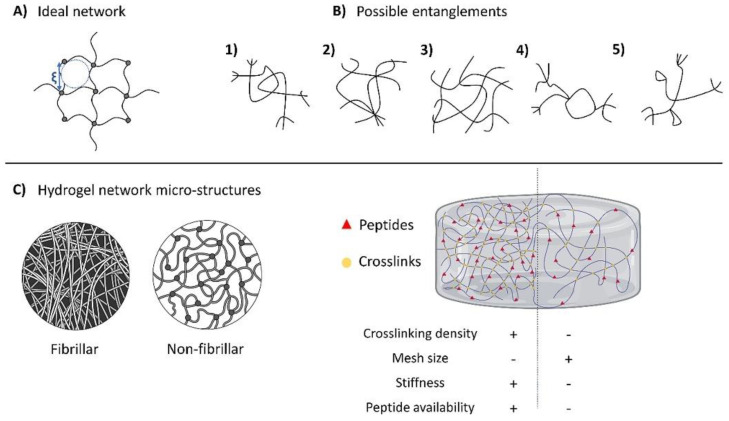
Structure of hydrogels where (**A**) ideal network, (**B**) entanglements, and (**C**) hydrogel network micro-structures. Reprinted from Solbu et al. [75].

Hydrogels are porous three-dimensional networks with high water content capacity due to the presence of hydrophilic groups attached to their polymer structure, e.g., hydroxyl, amine, carboxyl, and so on (Figure 2) [14,24,70]. The space can incorporate molecules (e.g., drugs or bioactive compounds) and other solvents (PBS buffer) and can be used for biomedical applications such as tissue engineering, wound healing, and drug delivering [71,72]. Also, hydrogels are distinguished by having interconnected polymeric networks that absorb water in large quantities without decomposing their structure [73,74].

Hydrogels possess a high porosity, suitable pore size, elasticity, biocompatibility, and adjustable physical, chemical, and biological properties [22,76]. An injectable hydrogel is a biomaterial that can be injected as a liquid into the human body and then forms an in situ solid hydrogel due to the increase in temperature. However, injectable hydrogels are not only all those that gel once they have been injected into the human body. Hydrogels with shear-thinning and self-healing properties are also classified as injectable biomaterials [23,77,78].

### 3.1. Hydrogel Classification

Hydrogels are classified according to their polymer nature, crosslinking method, composition (homo or copolymeric), electrical charge, and size.

#### 3.1.1. Natural Hydrogels

Natural polymers are extraordinary polymers for hydrogel production, since they have chemical structures comparable to the extracellular matrix of human tissues [73]. By origin, natural polymers display suitable biocompatibility, environmental sensitivity, and abundant availability in nature. These polymers have natural binding sites responsible for enhanced interactivity between the cells and hydrogels, and they could also be modified to provide tunability. Despite these advantages, natural polymers are often associated with low stability, batch-to-batch variability, poor mechanical properties, and rapid degradation rates [3,79,80].

In this group, we can find chitosan, gelatin, cellulose and its derivatives, hyaluronan, agar, fibrin, collagen, etc. These polymers have functional groups that facilitate chemical modification, and the gelling of many natural polymers can be controlled by temperature and pH [81]. In the last year, decellularized tissues have been used to extract biological molecules such as collagen, peptides, and sulfated glycosaminoglycans with an ability to undergo in situ gelation [82,83,84].

#### 3.1.2. Synthetic Polymers

Synthetic polymers are human-made prepared through the polymerization of a monomer; they include polyvinyl alcohol, polyethylene glycol, polyethylene oxide, poly-2-hydroxyethyl methacrylate, poly-N-isopropyl acrylamide, polyacrylic acid, and polyacrylamide. They are stable and have higher mechanical strength than natural polymers [85,86,87]. Synthetic polymers have advantages related to their tunability and the optimization of their characteristics to obtain desirable physicochemical and mechanical properties, porosity, and mesh size. However, synthetic polymers have limitations, including the lack of cell adhesion sites, low biocompatibility, and toxic degradation products [79,88].

#### 3.1.3. Crosslinking Method

The crosslinking method is critical for the final physicochemical and mechanical properties as well as the stability of hydrogels. Physical hydrogels are formed by reversible physical interactions such as ionic interactions, hydrogen bonds, hydrophobic interactions, or crystal formation, and they can be destroyed by changing environmental conditions [2,27,77]. In contrast, in chemical hydrogels, the interactions between polymer networks are permanent due to chemical reactions such as radical polymerization, Michael addition, Schiff’s base reaction, or photo-polymerization [3,89]. While chemical crosslinking results in higher stability and mechanical strength of hydrogels, their implantation in tissue engineering is limited by the toxicity of chemical crosslinkers [43,90].

##### In Situ Physical Gels

Hydrogels produced in situ undergo a transition from a solution to a gel state, triggered by stimuli such as temperature, pH, or irradiation [91,92]. They can incorporate primary cells, stem cells, growth factors, and differentiating factors in situ in the matrix during the transition, leading to the formation of a three-dimensional (3D) scaffold for tissue engineering applications [23,93]. Other systems undergo gelation/solidification when the temperature decreases or have an inverse gelling property characterized by a lower critical solution temperature. In this case, the material undergoes a sol–gel transition and forms a solid polymer network. For biomedical applications, thermo-gelling injectable systems with a lower critical solution temperature around or below 37 °C would be ideal, as they would transform from a solution to a gel upon injection into a bodily cavity [57,94,95].

##### In Situ Chemical Gels

Chemical hydrogels are mainly formed by covalent bonds after specific chemical reactions. They can be prepared by using a hydrophilic monomer polymerized in the presence of a polyfunctional crosslinking agent or by the direct crosslinking of water-soluble monomers in the presence of a free-radical-generating initiator that can be activated by radiation (light, heat, etc.) or by chemical reactions (redox) [27,77]. Chemical crosslinking imparts mechanical integrity and degradation resistance to otherwise weak materials. Unlike preformed scaffolds, the crosslinking agent of the injectable gel cannot be washed away or quenched before implantation. For this reason, all reactants used must be non-toxic at the concentrations they are employed [57].

## 4. Repair of Nervous Tissue by Injectable Hydrogels

Injectable hydrogels are biomaterials biocompatible with high water content, tissue-like mechanical properties, and the ability to deliver regenerative factors, including proteins, small molecules, and even living cells [23,96,97]. This class of biomaterials can remain in the injury site after gelation, maintaining its biological properties for a specified time. Simultaneously, the healing process occurs, so it is necessary to develop hydrogel systems that solidify naturally (e.g., due to temperature and pH changes) without any chemical manipulation that would change the material’s structure [91].

### 4.1. Nervous System

Injury to the nervous system can lead to a decrease in sensory and motor function, paralysis, or death. In the last year, hydrogels have been used to promote neural regeneration and functional recovery and to address both peripheral and central nervous system injuries [98,99].

In this sense, in vitro and in vivo studies have potentially been conducted using injectable hydrogels for neural tissue engineering. Bousalis et al. [100] described that injectable hydrogels can mimic the native nerve extracellular matrix, displaying suitable properties for minimally invasive applications and biological conditions for neural cells.

**Figure 3:**
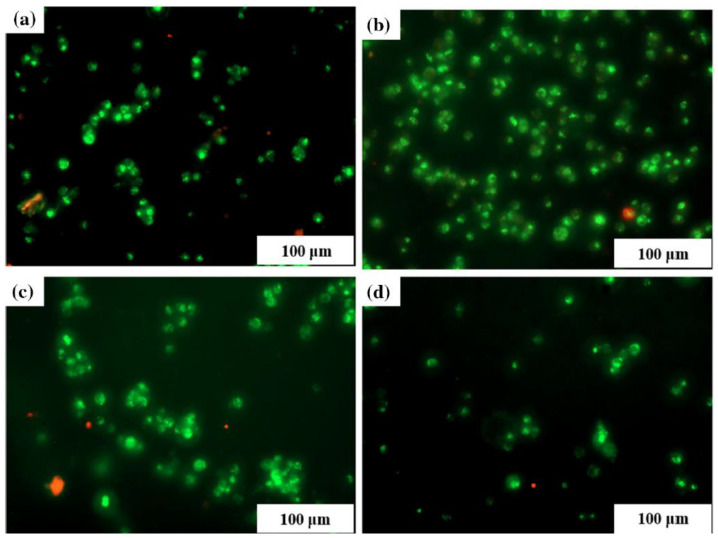
Live–dead assay of hyaluronic acid (HA)–puramatrix (RADA) hydrogels at 7 days where (**a**) HA-1, (**b**) HA-1-RADA-1, (**c**) HA-1-RADA-5 and (**d**) HA-1-RADA-10. The green color indicates viable cells and the red color indicates dead cells. Reprinted from Abbasi Aval et al. [101].

Abbasi Aval et al. [101] developed a thermosensitive hyaluronic acid–Puramatrix^TM^ peptide gelled at a physiological temperature. The porous hydrogel displayed an aligned unidirectional fibrous structure with elastic and high swelling behavior (100%). The hydrogel supported the viability of human neuroblastoma cells, which were uniformly dispersed through the polymer structure (Figure 3).

Mozhdehbakhsh Mofrad and Shamloo [102] produced a thermoresponsive chitosan hydrogel with conductive aligned nanofibers composed of polycaprolactone/gelatin/single-wall carbon nanotubes. The biodegradable hydrogel displayed a porous structure with interconnected pores with a pore diameter and porosity of 26.3–50.9 µm and 68.3–78.7%, respectively. The hydrogel was not cytotoxic towards human glioblastoma cells, with cell viability values higher than 70%, verified using MTT assay. Also, the hydrogel promoted cell adhesion to the microstructure, where cells displayed elongated and dense populations with reduced distances.

**Figure 4:**
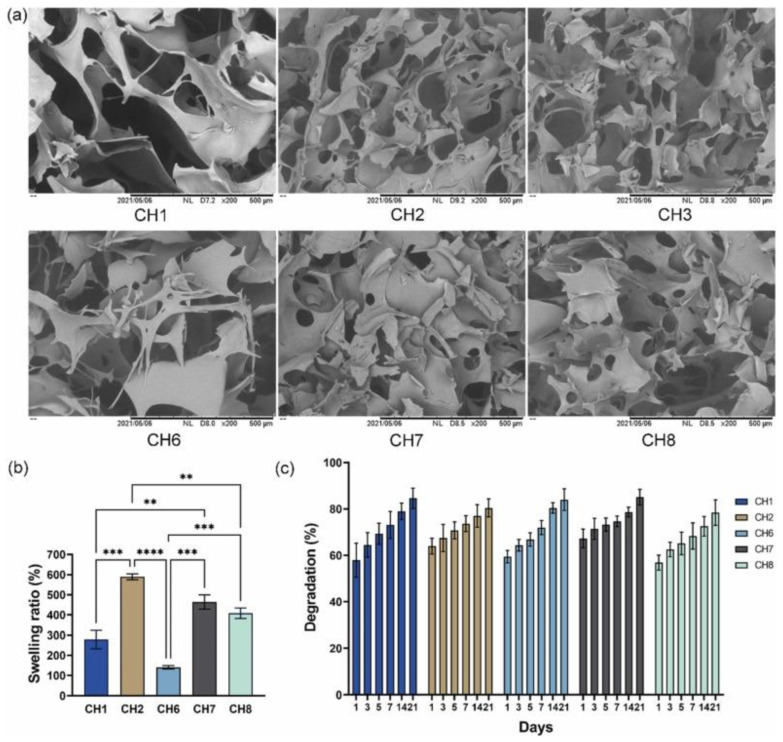
(**a**) Scanning electron microscopy (SEM) images, (**b**) swelling ratio (%), and (**c**) enzymatic degradation (%) of chitosan/β-GP hydrogels. Data was analyzed by one-way ANOVA and Tukey’s test where *p* ≤ 0.01 **, *p* ≤ 0.001 ***, and *p* ≤ 0.001 ****. Reprinted from Bhuiyan et al. [103], copyright 2023, with permission of Elsevier.

Bhuiyan et al. [103] produced a thermoresponsive chitosan hydrogel using β-glycerophosphate as the crosslinker agent. The hydrogel displayed biodegradable properties with a porous microstructure, mean pores size between 25 to 115 µm, and a high swelling ratio between 140.2 and 589% (Figure 4).

The hydrogel was seeded with rat pheochromocytoma cells, which were viable after 24 h of incubation. Similar results were reported by Furlani et al. [104] (astrocytes), Nguyen et al. [105] (human mesenchymal stem cells and human-induced pluripotent stem cell-derived neural stem cells), Olguín et al. [106] (transplantable rat pheochromocytoma), and Farrell et al. [107] (embryonic mouse dissociated brain cells).

Moreover, Nguyen et al. [105] produced an enzymatically crosslinked injectable hydrogel composed of hyaluronic acid, dopamine, and 3-(4-hydroxyphenyl) propionic acid. This hydrogel displayed a porous microstructure with a mean pore size between 50 and 300 μm. Human neural stem cells derived from induced pluripotent stem cells were seeded into the hydrogel, displaying a round shape morphology.

#### 4.1.1. Peripheral Nerve Injuries

The conventional treatment for peripheral nerve injuries consists in the use of autologous nerve transplantation, but it requires numerous surgical treatments and is affected by donor limitation, loss of nerve function, and scar formation. Tissue engineering allows to produce nerve conduits biocompatible with soft biomechanical structures and suitable flexibility. Also, scaffolds should mimic the natural extracellular matrix surrounding the nerve [59,108].

Recent in vivo reports show that central nerves to which injectable hydrogels are applied have a great potential recovery ability (Table 1).

Xu et al. [109] produced injectable chitosan graft–hyaluronic acid hydrogels loaded with nerve growth factors. The hydrogels gelled under physiological pH conditions and the gelation time decreased with increasing molar ratio of chitosan/hyaluronic acid. The hydrogels displayed continuous porous network structures from 82 to 87.12% with mean pore sizes between 42.19 and 73.53 µm. The hydrogels absorbed suitable water concentrations, displaying values between 13 and 18 *w*/*w*, and the release profile of nerve growth factors was about 70 and 98% within 56 days of incubation. RSC96 Schwann cells were cultured into the hydrogels for 3 days, displaying higher cell numbers than the control. Also, hydrogels loaded with nerve growth factors enhanced cell adhesion and proliferation. In vivo studies confirmed that rats walked as normal and the surgical side of the hindlegs did not vary from the unoperated side within three months of the operation. Also, rats implanted with injectable hyaluronic acid–chitosan/neural growth factors exhibited a suitable sciatic function index, which is indicative of motor recovery.

**Figure 5:**
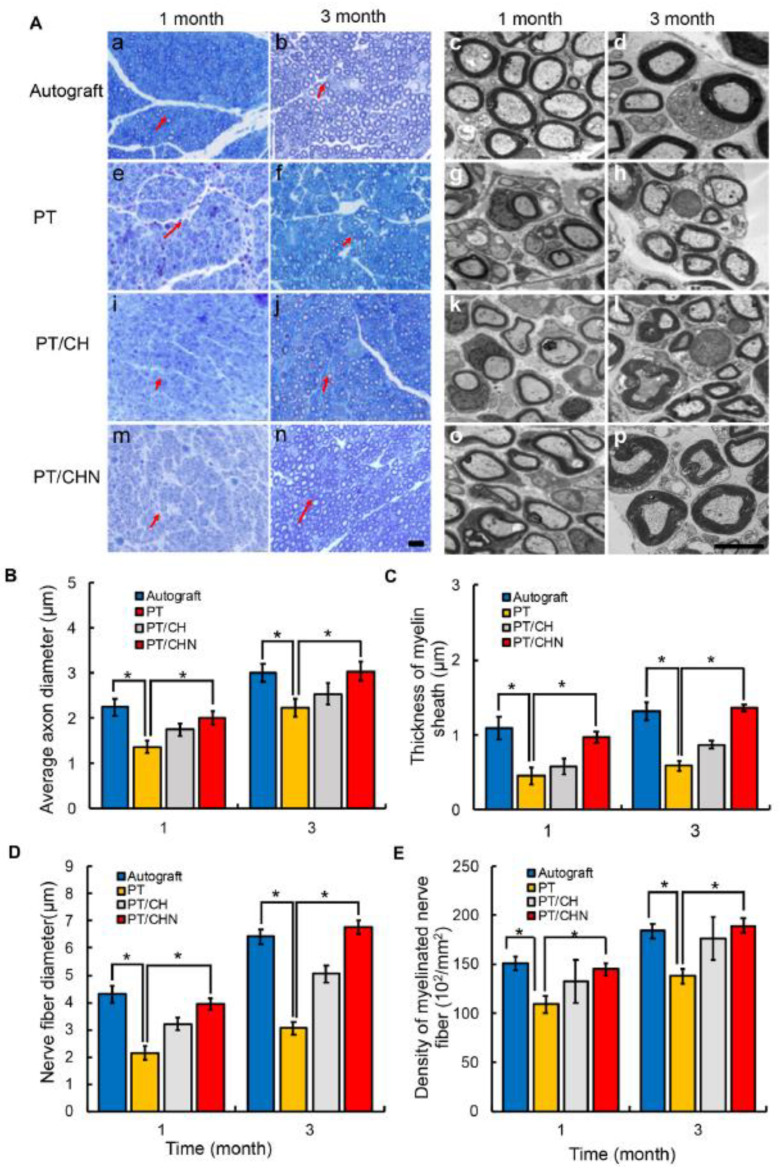
(**A**) Cross-sections of regenerated nerves taken from nerve conduits implanted in rats after 1 and 3 months. Red arrows show nerve fibers. (**a**–**d**): Autograft group; (**e**–**h**): Poly(D, L-lactic acid)/β-tricalcium phosphate nerve conduits group; (**i**–**l**): Poly(D, L-lactic acid)/β-tricalcium phosphate nerve conduits/hyaluronic acid-chitosan group; (**m**–**p**): Poly(D, L-lactic acid)/β-tricalcium phosphate nerve conduits/hyaluronic acid-chitosan/ nerve growth factor group. (**B**) Axon diameters of regenerated myelinated nerve fibers. (**C**) Thicknesses of regenerated myelinated sheaths. (**D**) Diameters of regenerated nerve fibers. (**E**) Densities of regenerated myelinated nerve fibers. Data was analyzed by one-way ANOVA where *p* < 0.05 *. Reprinted from Xu et al. [109], copyright 2022, with permission of Elsevier.

Figure 5 displays cross-sections of regenerated nerves taken from nerve conduits implanted in rats after 1 and 3 months. The results demonstrated that injectable hyaluronic acid–chitosan/neural growth factor implanted in rats induced a higher maturity and number of nerve fibers than the control group. Also, regenerated nerve fibers presented a uniform distribution three months after the implantation.

Therefore, injectable hydrogels containing neural growth factors enhanced the regeneration of deteriorated nerves. The nerves regenerated by injectable hydrogels were like those in the autograft group, indicating suitable nerve regeneration.

**Figure 6:**
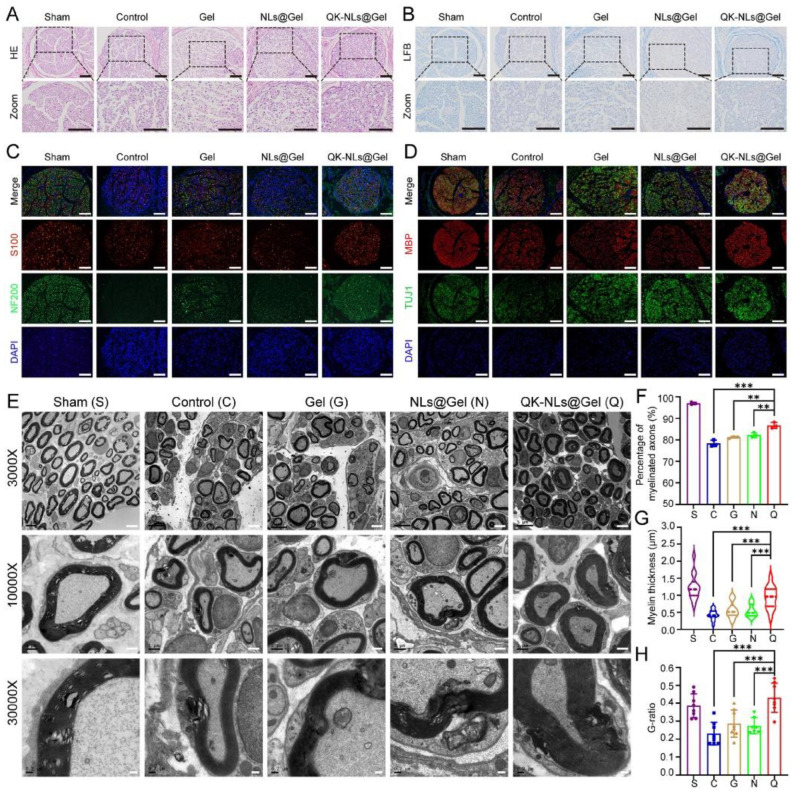
Middle cross-section of regenerated nerves at day 28 post-surgery. (**A**) Hematoxylin and eosin (HE) staining, (**B**) Luxol fast blue staining, (**C**) immunofluorescence staining of S100 and neurofilament-200, (**D**) immunofluorescence staining of myelin basic protein and beta III Tubulin, (**E**) TEM images of transverse sections of regenerated nerve fibers, (**F**) myelinated axons from TEM observation, (**G**) myelin thickness from TEM observation, and (**H**) axon-to-fiber diameter of regenerated nerves from TEM observation. *p* < 0.01 **; *p* < 0.001 ***. Reprinted from Xu et al. [110], copyright 2023, with permission of Elsevier.

Xu et al. [110] developed injectable hydrogels composed of methacrylated gelatin loaded with vascular endothelial growth factor/mimetic peptide nanoliposomes. The hydrogels were crosslinked by photo-crosslinking using UV-light. PC-12 and RSC96 cells were seeded into the hydrogels, displaying high viability (higher than 80%) within 3 days of incubation. The authors evaluated the neovascularization process during the nerve regeneration process in rats. The results displayed high micro-vessel densities 7 days after implantation (66 micro-vessels/field), while two critical angiogenetic markers favoring revascularization (Hif-1 α and VEGFR2) were identified. Figure 6 displays the middle cross-section of regenerated nerves at day 28 post surgery.

The authors evaluated segments of regenerated nerves in the group where the hydrogels had been used. The results showed no evident necrosis or scar tissue 28 days after the surgery (Figure 6A). Also, the axons exhibited suitable remyelination and regenerated axons were surrounded by thick, transparent, and electron-dense myelin sheaths. Fascinatingly, the hydrogel composed of methacrylate gelatin loaded with vascular endothelial growth factor/mimetic peptide nanoliposomes prompted the maturation of regenerated axons, indicating suitable axon regeneration and remyelination of injured nerves.

Liu et al. [111] produced injectable chitosan hydrogels loaded with black phosphorus nanosheets and tazarotene. The hydrogels were photo-crosslinked by UV irradiation at 365 nm. The porous hydrogels with a mean pore size from 80 to 160 μm promoted the viability and differentiation of PC12 cells within seven days of incubation. The in vivo study indicated that the hind limb motor function of rats was recovered (BBB score 9.38), which was verified with motor footprints.

**Figure 7:**
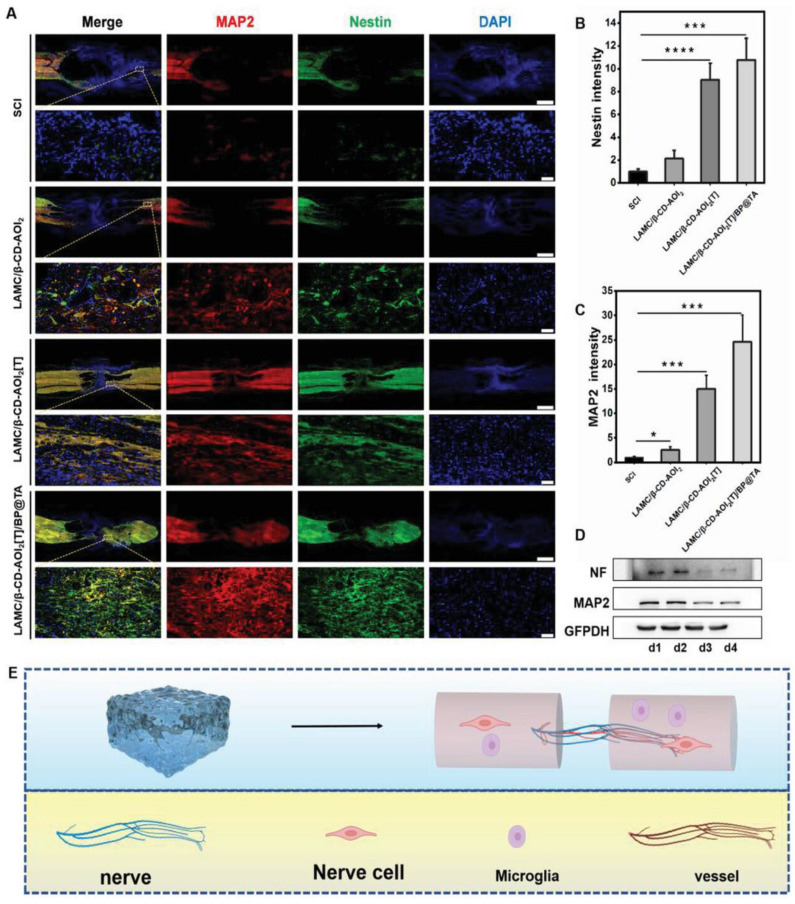
Hydrogel implantation promotes nerve regeneration. (**A**) Immunohistofluorescence images of longitudinal spinal cord sections from rats. (**B**) Quantitative analysis of Nestin, (**C**) Quantitative analysis of MAP2, (**D**) Protein expression of VEGF and CD31 of different groups and (**E**) Scheme of hydrogel-releasing drug and conductive black phosphorus promoting vascular regeneration. * *p* < 0.05, *** *p* < 0.001, **** *p* < 0.0001. Reprinted from Liu et al. [111], copyright 2024, with permission of Elsevier.

Figure 7 indicates that distal nerves in the groups where hydrogels had been implanted conserve their normal functionality and survival, since Microtubule-Associated Protein 2 and Nestin markers staining on the distal nerves were observed to be more intense (Figure 7A).

These results were confirmed by Western blot analysis, where protein expression levels were increased according to the marker (Figure 7D).

#### 4.1.2. Spinal Cord Injuries

The conventional treatment procedure for spinal cord injuries consists of hormone shock, surgical decompression, spinal fixation, and rehabilitation. However, these methods have not produced positive results for treating spinal cord injury [114]. Tissue engineering allows for the production of biocompatible and biodegradable scaffolds which in spinal cord injury would allow for the encapsulation of cells, drugs, or bioactive molecules with injectable and self-healing properties and their insertion into the damaged tissue. Also, scaffolds must possess suitable mechanical properties that support cells and tissues and promote controlled drug delivery [115]. These scaffolds must provide hydrogels with three-dimensional polymer structures for neuronal regeneration and axonal extension that would help promote cell adhesion, growth, proliferation, and migration [56,98].

Table 2 displays the recent advances in injectable hydrogels for spinal cord injury.

**Figure 8:**
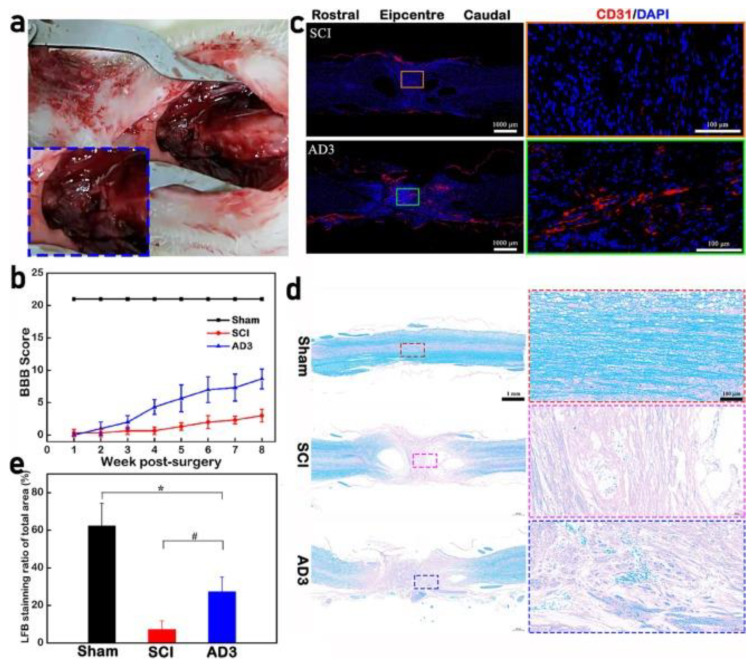
Promotion of angiogenesis process by hyaluronic acid-based hydrogels after spinal cord injury. (**a**) Laminectomy and injected the hydrogel in the lesion area, (**b**) behavior assay of BBB scores, (**c**) spinal sections staining CD31 (red) and DAPI (blue) from spinal cord injury and hydrogel-treated rats at 56 days post-injury, (**d**) luxol fast blue staining of longitudinal sections at 56 days post-injury, and (**e**) quantification of luxol fast blue staining ratio of the total spinal cord. * *p* < 0.05 compared with Sham and # *p* < 0.05 compared with spinal cord injury. Reprinted from Li et al. [124], copyright 2022, with permission of Elsevier.

Li et al. [124] produced an injectable and self-healing hyaluronate hydrogel by chemical crosslinking using Schiff’s base reaction. The sol-gel transition of hydrogels was reached within 50 s, displaying an elastic behavior. The hydrogel had a microporous structure with a mean pore size between 20 and 180 μm and a high swelling ratio from 15 to 45 g/g at pH 7.4 during incubation. The hydrogel displayed suitable self-healing properties and biocompatibility. In this sense, neural stem cells were seeded into the hydrogel within 48 h of incubation, where cells were viable with a regular shape attached to the hydrogel. The authors carried out immune staining analysis, demonstrating the identification of neural biomarkers such as Tuj1, which is related to neuron-specific microtubule elements. These results indicated that the hydrogel could enhance neural activity and neural differentiation. Figure 8 shows the promotion of the angiogenesis process by hyaluronic acid-based hydrogels after spinal cord injury.

The authors carried out a laminectomy by removing the spinal tissue of rats. Then, the injectable hydrogel was deposited into the damaged region. The authors analyzed the influence of the hydrogel on motor function recovery, which was quickly achieved within 8 weeks post surgery (Figure 8b). Figure 8c reveals the presence of the CD 31 biomarker, indicative of vascular regeneration and newly formed micro-vessels. Also, the hydrogel induced remyelination (blue point) around the treated cavity, which is critical for spinal cord injury repair.

**Figure 9:**
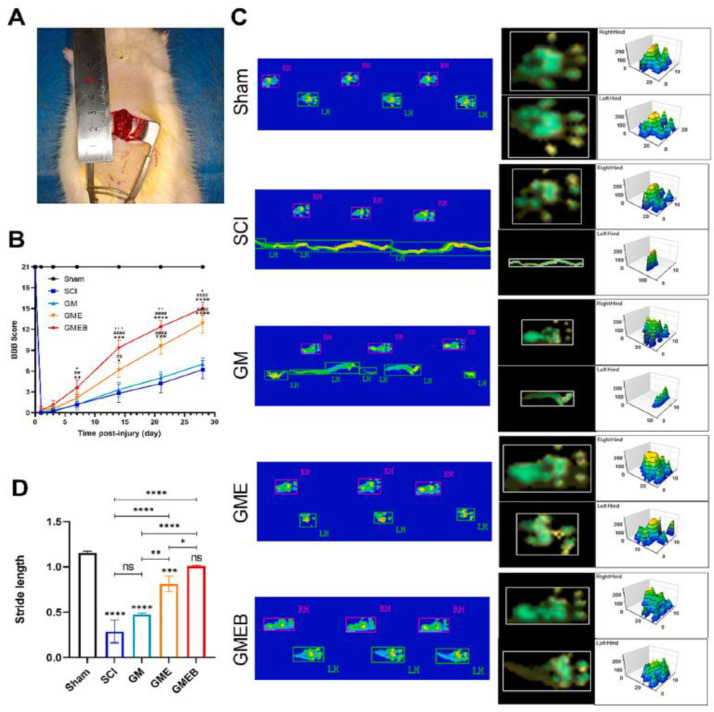

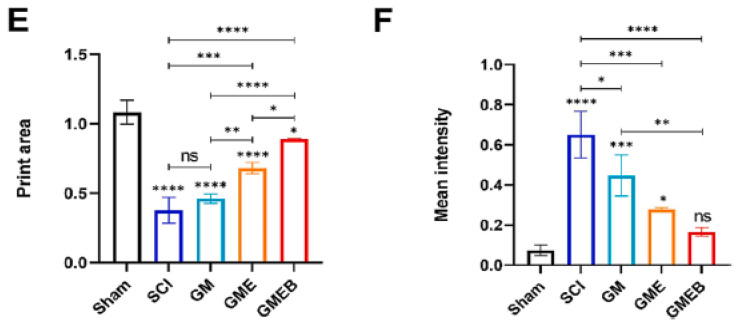
Recovery of motor function after spinal cord injury in rats. (**A**) Rat left spinal cord hemisection injury model, (**B**) Left hindlimb locomotor recovery by the BBB scale. Rats treated with the GMEB hydrogel, (**C**) Footprints used to analyze the recovery of hindlimb motor function, (**D**) Quantification of the stride length, (**E**) print area, (**F**) and mean intensity (**F**) of the left hindlimb in each group 4 weeks after operation. ^+^, * *p* < 0.05, ^++^, ##, ** *p* < 0.01, ^+++^, *** *p* < 0.001, ####, **** *p* < 0.0001, ns = not significant. Reprinted from Wang et al. [131], copyright 2023, with permission of Elsevier.

Wang et al. [131] developed an injectable gelatin methacryloyl hydrogel by photo-crosslinking, using berberine, a natural alkaloid, as the carrier. The hydrogel displayed an elastic behavior and a porous structure, with a mean pore size between 116.4 and 127.2 μm and porosity values between 41.49 and 38.93%. Also, over 80% of the hydrogel had biodegraded within 21 days of incubation at 37 °C. Berberine molecules were successfully loaded into the hydrogel, displaying a sustained release of about 80% by day 14 of incubation. The authors induced a lesion on the spinal cords of rats and implanted the hydrogel, loaded with berberine, to evaluate the recuperation of motor function (Figure 9A). The hydrogel promoted the recovery of motor function in rats at 1 week post surgery. Fascinatingly, the rats displayed sustained palmar weight-bearing movement and coordinated anterior and hind limb movements at 28 days post surgery (Figure 9B).

Figure 9C shows the footprint analysis of the rats and demonstrates the previous results. Rats treated with the hydrogel loaded with berberine showed coordinated movement of both the front and hind limbs (Figure 9C). Figure 9D–F show no statistically significant differences between the stride length and mean intensity in the experimental and sham surgery groups. Rats implanted with the hydrogel showed a substantial reduction in local hyperplasia, with suitable organization of the regenerated tissues. Therefore, the hydrogel modulated the pathophysiological processes of spinal cord injury, restrained aberrant tissue growth, and inhibited the spread of damage.

Luo et al. [134] produced a hydrogel loaded with curcumin composed of 9-fluorenylmethoxycarbonyl-glycine/chitosan conjugated using 1-ethyl-3-(3-Dimethylaminopropyl) carbodiimide/N-hydroxysuccinimide. The polymer solution showed reversible properties with suitable injectability, as well as self-healing properties. Two hydrogel samples were integrated without supplementary stimuli. At low strain values, the hydrogel showed a predominantly elastic behavior (from 0.1 to 100%), while the crossover point occurred at around 600%. Markers CD68 and ARG1 (anti-inflammatory phenotype) were identified within the hydrogel in the lesion site 2 weeks after surgery.

**Figure 10:**
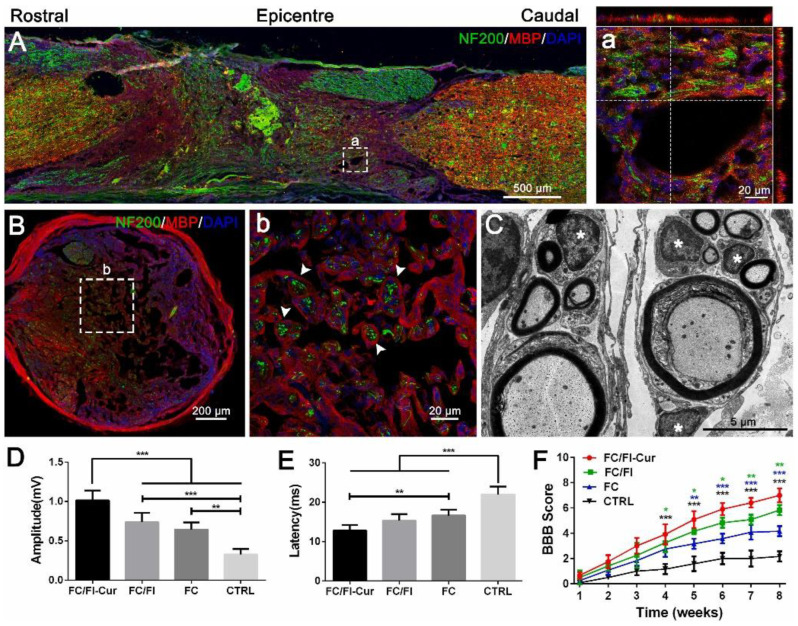
Remyelination and functional recovery 2 months after implantation of hydrogel loaded with curcumin following spinal cord injury. (**A**) Remyelination in the graft, (**a**) nerve fibers, (**B**), (**b**) transversal section in the lesion, (**C**) regenerated tissues in cross sections of the lesion, (**F**) BBB open-field walking scale of rats, (**D**) amplitude and (**E**) latency of motor evoked potential. * *p* < 0.05, ** *p* < 0.01, *** *p* < 0.001. Reprinted from Luo et al. [134].

Figure 10 displays remyelination and functional recovery 2 months after the implantation of hydrogel loaded with curcumin following spinal cord injury.

Figure 10A displays intimate contact between the neural filaments and the myelin sheath (Figure 10A), while Figure 8b shows images of a transversal section where the MBP+ myelin sheath displays entire “O” rings, encircling neurofilament-immunoreactive axons, in which hydrogel-induced remyelination is completed. Figure 10C describes typical layered myelin sheets surrounding the axoplasm, where neurofilaments and vesicles can be located. In this context, the goal of myelination is restoring conduction activity for signal transduction [134].

#### 4.1.3. Brain Injury

Kornev et al. [27] describe several requirements that biodegradable scaffolds must have: injectability, shear-thinning and self-healing, biocompatibility, low cytotoxicity, non-immunogenicity, non-mutagenicity, and promotion of cell proliferation, migration, and differentiation. Also, scaffolds must be soft and allow for the encapsulation of cells, drugs, or bioactive molecules as well as promote controlled drug delivery [115,137]. Table 3 displays the advances in injectable hydrogels for brain injury.

Zhang et al. [146] produced an injectable antioxidant gallic acid–grafted hyaluronic acid hydrogel blended with hyaluronic acid–tyramine. The hydrogel was enzymatically crosslinked using horseradish peroxidase and hydrogen peroxide produced by oxidase of D-galactose catalyzed by galactose oxidase.

The biodegradable hydrogel displayed elastic behavior and absorbed high water concentrations (<90%), with a gelation time between 1 and 8 min which was dependent on hyaluronic acid concentration. Also, the hydrogel displayed a porous and interconnected structure with a mean pore size of 346 μm, which would help in the gases diffusion, nutrients, and waste. The hydrogel provided antioxidant activity by a scavenging effect against DPPH radicals, while also providing suitable viability towards mouse neuroblasts at high hyaluronic acid concentrations (0.5 and 0.75%) after 48 h of incubation. The hydrogel displayed hemocompatibility, with a hemolysis ratio lower than 5%. The hydrogel implanted by subcutaneous injection into rats did not cause an inflammatory reaction. The in vivo study exhibited decreased malondialdehyde concentration and increased glutathione expression in the lesion area 21 days after implantation, which is related to the regulation of detoxification and antioxidant, anti-inflammatory, and cytoprotective activities.

**Figure 11:**
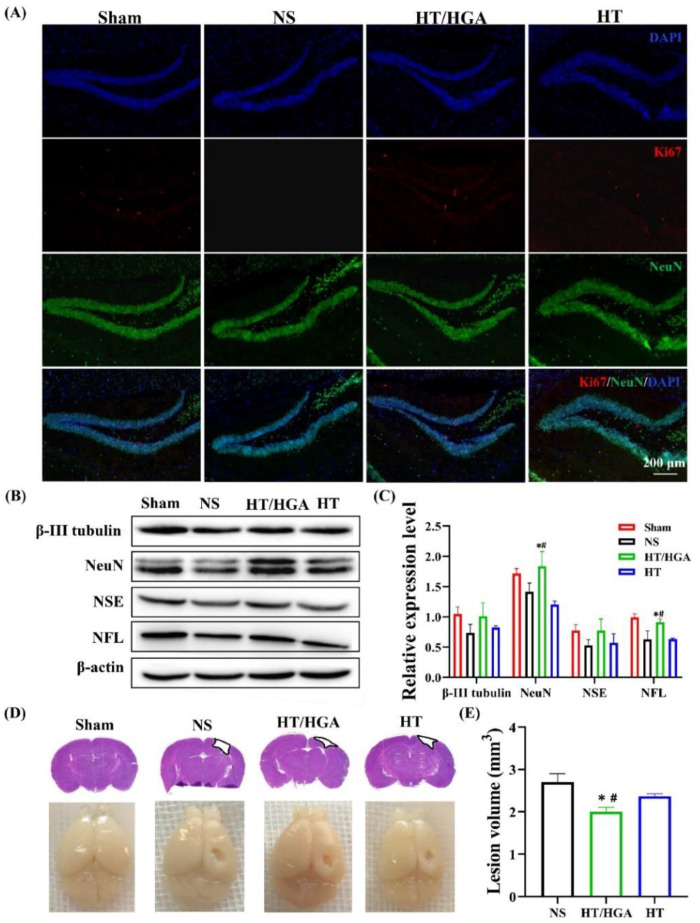
Neurogenesis and brain tissue remodeling after treatment for 21 days. (**A**) Immunofluorescence staining of NeuN and Ki67, (**B**) western blot and (**C**) relative expression of β-III tubulin, NeuN, NSE and NFL, (**D**) H&E staining and imaging of TBI mice brain, (**E**) brain lesion volume. *, # *p* < 0.05. Reprinted from Zhang et al. [146].

Figure 11A displays immunofluorescence images that analyze neurogenesis in the rat hippocampus. Ki67 and NeuN were used as proliferation- and neuron-specific markers. Rats treated with the hydrogel displayed high Ki67 and NeuN expression 21 days after implantation. The use of the hydrogel promoted the expression of neuron-related proteins (β-III tubulin, NeuN, NSE and NFL), which was corroborated by Western blot analysis (Figure 11B,C). Figure 11D demonstrates suitable brain structure recovery and reconstruction using hydrogel implantation in rats, which enhanced neural cell viability and neurogenesis.

Nourbakhsh et al. [148] produced an injectable hydrogel based on Pluronic-chitosan/aniline-pentamer containing an angiogenic factor. The hydrogel gelled between 4 and 7 min, which increased with decreasing Pluronic concentration. Also, the hydrogel displayed a high degradation rate (10–40% within 40 days of incubation) and high swelling behavior (500–800%) in PBS at 37 °C. Furthermore, the hydrogel displayed antibacterial activities against *Escherichia coli* and *Staphylococcus aureus.* The hydrogel was seeded with pheochromocytoma cells, which showed good adhesion, while cell viability increased with time; the highest viability was obtained after 5 days of incubation.

Nourbakhsh et al. [148] demonstrated that following the injection of the hydrogel containing vascular endothelial growth factor, brain infarct volume was reduced in comparison with the control group. Also, rats treated with vascular endothelial growth factor-containing hydrogel showed a smaller ischemic area compared with those treated with vascular endothelial growth factor alone. Also, hydrogels loaded with growth factor caused improved hippocampal-dependent learning and memory performance in rats.

## 5. Conclusions

Injectable hydrogels are a potential biomaterial for nervous tissue repair. Hydrogels possess fascinating properties, such as porosity, interconnectivity, suitable mechanical properties like those of nervous tissue, swelling behavior, and biocompatibility. In this review, we described and discussed recent advances in injectable hydrogels for in vivo nervous tissue repair in brain, peripheral nerve, and spinal cord injury. In the literature, we found that injectable hydrogels can enhance their biological properties by encapsulating drugs or bioactive molecules which are crucial for nervous tissue regeneration. However, most of the investigations were focused on the synthesis of complex injectable hydrogels, where several polymers were conjugated to obtain the desirable physical, chemical, structural, and biological properties. As we all know, it is important to carry out simple synthesis processes with the lowest number of stages possible, as with this, it is possible to achieve a reduced impact on the environment, generating less chemical waste.

Due to the complexity of nervous tissue, the area of biomaterials has some challenges to address to improve the properties of injectable hydrogels. In this way, they will potentially improve the pathologies of nervous tissue. 

In conclusion, the injectable hydrogels reviewed and discussed in this brief review display potential for the repair and regeneration of the brain, peripheral nerves, and spinal cord.

## Figures and Tables

**Table 1:** Injectable hydrogels for peripheral nerve injuries.

Polymer Blend	Crosslinking Method	In Vivo Model	In Vitro Model	Biological Results	Ref.
Hyaluronic acid/chitosan bioconjugate using EDC/NHS	Physical crosslinking (in situ gelation)	Rats	Rat Schwann cells Bone marrow-derived MSCs PC12 cells	Biodegradability and biocompatibility. Proliferation differentiation and axonal growth. Functional recovery of nerve defects. Axon regeneration and myelination.	[109]
Methacrylated gelatin loaded with peptide/VEGF/nanoliposomes	Photocrosslinking by UV irradiation	Rats	Human primary endothelial cells RSC96 cells PC-12 cells	Viability and proliferation. Revascularization, pro-healing, remyelination, and axon regeneration. Functional restoration and reversal of denervated muscle atrophy.	[110]
Chitosan/lipoic acid bioconjugate using EDC/NHS	Photocrosslinking by UV irradiation	Rats	PC-12 cells	Non-cytotoxicity and neurite extension. Neural cell differentiation. Recovery of motor function. Endogenous angiogenesis and neurogenesis. Vascular regeneration.	[111]
Decellularized extracellular matrix (bone, liver, and intestine)	Physical crosslinking by neutralization	Rats	Schwann cells Dorsal root ganglion cells	High metabolic activity. Neurite extension and axonal regeneration.	[112]
Hyaluronic acid/pamidronate–magnesium	Physical crosslinking (in situ gelation)	Rats	Dorsal root ganglion neurons	Neurite outgrowth. Axon regeneration and remyelination. Peripheral nerve regeneration. Functional recovery.	[113]

Abbreviations. EDC: 1-Ethyl-3-(3-Dimethylaminopropyl) Carbodiimide, NHS: N-hydroxysuccinimide, MSCs: mesenchymal stem cells, VEGF: vascular endothelial growth factor, UV: ultraviolet.

**Table 2:** Recent advances in injectable hydrogels for spinal cord injury.

Polymer Blend	Crosslinking Method	In Vivo Model	In Vitro Model	Biological Results	Ref.
Hyaluronan and methylcellulose	Physical crosslinking	Rats	Neural stem cells	Cell viability and proliferation. Recovery of locomotor functions. Functional tissue repair. Reduction in lesion size and inflammatory response. Increased sparing of perilesional host neurons and oligodendrocytes.	[116]
Chitosan	Physical crosslinking by neutralization using ammonia	Rats	ND	Axonal regrowth. Prevention of mature scar formation. Promotion of remyelination and reconstitution of spinal tissue and vasculature. Immune-modulatory action. Recovery of locomotor functions.	[117]
Norbornene/hyaluronic acid	Chemical crosslinking by redox radical formation using tetramethylethylenediamine	Goat	Goat neural stem cells	Restored disc mechanics in a degenerated disc. Maintained structural integrity of the disc. Increase in proteoglycan and collagen concentration in the nucleus pulposus. Suitable treatment of degenerative disc disease.	[118]
Poly(D,L-lactic acid-co-trimethylene carbonate), gelatin, and poly(ethylene glycol) diacrylate	Photocrosslinking by UV irradiation	Mouse	Embryonic stem cells	Non-cytotoxicity and cell differentiation. Recovery of spinal cord tissue and decrease in the formation of scar tissue. Functional neural regeneration and decreased neuroinflammation. Locomotor functional recovery.	[119]
Hydroxyphenyl derivative of hyaluronic acid (HA-PH) AND arginine-glycine-aspartic acid (RGD)	Chemical crosslinking	Rats	Schwann cells	Cell viability and cell adhesion. Increased axonal growth. Locomotor and respiratory functional recovery.	[120]
Acellular nerve scaffold containing collagen and sulfated glycosaminoglycans	Physical crosslinking	Rats	Schwann cells	Increased axonal coverage after 8 weeks. Cell attachment and survival in vitro. Locomotor and respiratory functional recovery. Increased axonal growth and decrease in astrocytic scarring.	[121]
Methylcellulose/hyaluronan/peptides	Physical crosslinking by hydrophobic interactions	Rats	Neural stem cells	Promotion of survival in stem cells and in vivo model. Recovery of motor function.	[122]
Glycol chitosan/oxidized hyaluronate	Physical crosslinking by self-assembly and hydrophilic interactions	Rats	ND	Improved histopathological damage to spinal cord. Reduced expression levels of pro-inflammatory cytokines.	[123]
Hyaluronic acid/3,3′-dithiobis (propionyl hydrazide)	Chemical crosslinking by Schiff’s reaction	Rats		Self-healing ability and injectability. Good viability and non-cytotoxicity. Improved motor function recovery. Promotion of angiogenesis, remyelination, and neural regeneration.	[124]
Chitosan/hydroxyethylcellulose	Physical crosslinking using β-glycerol phosphate	Rats	Human adipose-derived stem cells	Promotion of cell growth and viability. Regeneration of injured spinal cord tissue. Cell proliferation in injured tissue. Improvement in locomotor recovery.	[125]
Silk fibroin/polydopamine	Physical crosslinking	Rats	Primary hippocampal neuron L929 cells	No cytotoxicity. Increase in axon length and cell density. Expression level of neuritis-related protein. Promotion of spinal cord injury repair. Capacity for inhibiting scar formation.	[126]
Sodium alginate, poly (lactic-co-glycolic acid), Resomer^®^, polyvinyl alcohol.	Physical crosslinking by ionic gelation D-gluconate)	Rats	ND	Recovery of locomotor function. Regeneration of damaged neurons and axons. Reduction in the activity of inflammatory cells. Reduction in formation of fibrotic scar tissue.	[127]
Chitosan	Physical crosslinking using β-glycerol phosphate	Mice	Mesenchymal stem cells	Antioxidant capacity by reducing reactive oxygen species. Cell viability and biodegradability. Promotion of an adequate environment for cell survival and biointegration. Reduction in the formation of glial scars.	[128]
Sodium carboxymethylcellulose and chitosan	Physical crosslinking	Rats	Neural stem cells	Non-cytotoxicity. Cell differentiation, proliferation, and viability. Improved mitochondrial dysfunction. Promotion of neurite outgrowth and neuronal maturation. Promotion of motor and urinary recovery.	[129]
Laminin, isoleucine-lysine-valine-alanine-valine peptides	Physical crosslinking	Rats	Dorsal root ganglia cells	Axon preservation and astrogliosis reduction. Minimal inflammation. Functional locomotor recovery.	[130]
Gelatin methacryloyl	Photo-crosslinking	Rats	Human umbilical cord mesenchymal stem cells and L929 cells	Cell differentiation, proliferation, and viability. Nerve regeneration, axon growth, and promotion of motor function recovery. Reduction in local hyperplasia, anti-inflammatory factors, and fibrosis.	[131]
Gelatin–g-polyaniline and sodium hyaluronate oxide	Physical crosslinking	Rats	Neural stem cells	Promotion of cell proliferation and neural differentiation. Reduction in the formation of glial scars and nerve regeneration. Locomotion recovery and nerve conduction function. Tissue reconnection and remyelination.	[132]
Borax–oxidized chondroitin sulphate–polypyrrole and gelatin	Double crosslinking (Schiff’s base, borate-diol ester bonds, and electrostatic interaction)	Rats	Neural stem cells	Good injectability and self-healing ability. Suitable biodegradation, cell viability, and hemocompatibility. Neuronal activity and neural differentiation. Axonal outgrowth, remyelination, and functional recovery. Promotion of endogenous neurogenesis and decreased glial scar formation in vivo.	[133]
Fluorenyl-chitosan and F-moc peptides	Physical crosslinking	Rats	Dorsal root ganglia cells	Good injectability and self-healing. Accelerated neurite outgrowth. Modulation of local anti-inflammatory reaction. Remyelination of regenerated nerves and functional recovery.	[134]
Polyethylene glycol and oxidized dextran	Physical crosslinking (in situ gelation)	Rats	Neural stem cells	Proliferation and neural differentiation. Inhibition of glial scar formation. Promotion of axonal regeneration and nerve circuit reconstruction. Optimal neural bridging network formation and locomotor improvement.	[135]
Dihydroxyphenylalanine-g-chitosan with peptide	Physical crosslinking (in situ gelation)	Rats	ND	Recovery of motor function. Recovery of sensory function. Bladder defect repair. Modulation of the immune response. Promotion of robust neural regeneration, synapse formation, and myelin regeneration.	[136]

Abbreviations: ND: not determined.

**Table 3:** Injectable hydrogels for brain injury.

Polymer Blend	Crosslinking Method	In Vivo Model	In Vitro Model	Biological Results	Ref.
Diblock copolypeptides bioconjugate using EDC/NHS	Physical crosslinking (in situ gelation)	Mice	Neural stem cells	Cell viability. Support of regrowth of host nerve fibers.	[138]
Keratin/PNIPAM	Physical crosslinking (in situ gelation)	Rats	ND	Reduction in intracerebral hemorrhage. Reduction in brain non-heme iron content, brain edema, and ROS level.	[139]
Hyaluronan, PEG, and chitosan	Physical crosslinking (in situ gelation)	Zebrafish and rats	Neural stem cells	Cell viability, adhesion, proliferation, and differentiation. Repair of the injured brain. Functional recovery.	[140]
Sodium alginate and hyaluronic acid	Physical crosslinking (ionic gelation)	Rats	Human umbilical cord mesenchymal stem cells	Cell growth, differentiation, and proliferation (in vitro). Recovered motor ability. Proliferation and regeneration of endogenous nerve cells (in vivo). Reduced inflammatory cells. Cell differentiation.	[13]
Choline–graphene oxide, polyacrylic acid	Physical crosslinking (in situ gelation)	Mice	Rat pheochromocytoma cells	Neurite branching. Biocompatibility. Cell growth. Full recovery from injury after 5–7 days.	[141]
Hyaluronan, methylcellulose and PLGA	Physical crosslinking (in situ gelation)	Rats	ND	Recovery of motor function. Neurogenesis and plasticity.	[142]
Hyaluronic acid/D-galactose	Enzymatic crosslinking using horseradish peroxidase and galactose oxidase	Mice	Bone mesenchymal stem cells	Cell viability and hemocompatibility. Recovery of neuromotor function. Recovery of learning and memory ability. Reduction in inflammatory response. Healing of damaged tissue.	[143]
Thiolated gelatin and polyethylene glycol diacrylate	Chemical crosslinking by Michael addition reaction	Mice	Mesenchymal stem cells	Cell adhesion and viability. Reduced brain damage and inflammation. Reduced neuron loss. Nerve functional recovery.	[144]
Phenol–chitosan	Chemical crosslinking using Pluronic F127	Rats	ND	Behavior improvement. Neural regeneration and angiogenesis.	[145]
Hyaluronic acid/galactose	Enzymatic crosslinking using horseradish peroxidase and galactose oxidase	Mice	ND	Cell viability and hemocompatibility. Reduction in oxidative stress. Enhanced neurogenesis and improved neural function recovery. Recovery of motor, learning, and memory ability.	[146]
Thiolated hyaluronic acid/thiolated collagen	Chemical crosslinking using polyethylene glycol diacrylate	Mouse and non-human primate	ND	Recovery of motor function. Promotion of axonal sprouting in motor system. Neurogenesis after stroke.	[147]
Pluronic-chitosan/aniline-pentamer	Physical crosslinking (in situ gelation)	Rats	Pheochromocytoma cells	Antibacterial capacity. Cell adhesion, non-cytotoxicity, proliferation, and viability. Reduction in infarct volume. Passive avoidance memory. Improve spatial learning and memory.	[148]
Hyaluronic acid	Physical crosslinking (in situ gelation)	Mice	ND	Long-term brain revascularization. Neurogenesis and axonogenesis. Functional recovery.	[149]
Collagen and polyethylene glycol	Physical crosslinking (in situ gelation)	Mice	Human neuro- blastoma cells and murine primary neurons	Cell viability, adhesion, and proliferation. No proinflammatory effects. No traumatic stress damage.	[150]
Phenylboronic acid/hyaluronic acid, dopamine/gelatin	Chemical, dynamic boronate ester bonds	Mice	Primary astrocytes	Cell viability and adhesion. Reduced glial scar formation. Ingrowth of neurons. Reduction in neural cell infiltration, astrogliosis, and glial scars. Favor close of the lesions.	[151]
Gelatin-hydroxyphenyl	Enzymatic crosslinking using horseradish peroxidase and galactose oxidase	Rats	Bone marrow stem cells	Biodegradability and non-cytotoxicity. Reduction in damage volume after traumatic brain injury. Accelerated healing process. Neurological function recovery.	[152]

Abbreviations. EDC: 1-Ethyl-3-(3-Dimethylaminopropyl) Carbodiimide, NHS: N-hydroxysuccinimide, PNIPAM: Poly(N-isopropylacrylamide), ROS: reactive oxygen species, PEG: poly-(ethylene glycol), ND: not determined.
